# Association between furosemide administration and outcomes in critically ill patients with acute kidney injury

**DOI:** 10.1186/s13054-020-2798-6

**Published:** 2020-03-04

**Authors:** Guang-ju Zhao, Chang Xu, Jian-chao Ying, Wen-biao Lü, Guang-liang Hong, Meng-fang Li, Bing Wu, Yong-ming Yao, Zhong-qiu Lu

**Affiliations:** 1 Emergency Intensive Care Unit, Emergency Department, The First Affiliated Hospital of Wenzhou Medical University, Wenzhou, 325000 People’s Republic of China; 2 Trauma Research Center, Fourth Medical of the Chinese PLA General Hospital, Beijing, 100048 People’s Republic of China

**Keywords:** Furosemide, Diuretic, Acute kidney injury, Critical care, Mortality

## Abstract

**Background:**

Although current guidelines for AKI suggested against the use of furosemide in AKI management, the effect of furosemide on outcomes in real-world clinical settings remains uncertain. The aim of the present study was to investigate the association between furosemide administration and outcomes in critically ill patients with AKI using real-world data.

**Methods:**

Critically ill patients with AKI were identified from the Medical Information Mart for Intensive Care (MIMIC)-III database. Propensity score (PS) matched analysis was used to match patients receiving furosemide to those without diuretics treatment. Linear regression, logistic regression model, and Cox proportional hazards model were used to assess the associations between furosemide and length of stay, recovery of renal function, and in-hospital and 90-day mortality, respectively.

**Results:**

A total of 14,154 AKI patients were included in the data analysis. After PS matching, 4427 pairs of patients were matched between the patients who received furosemide and those without diuretics treatment. Furosemide was associated with reduced in-hospital mortality [hazard ratio (HR) 0.67; 95% CI 0.61–0.74; *P* < 0.001] and 90-day mortality [HR 0.69; 95% CI 0.64–0.75; *P* < 0.001], and it was also associated with the recovery of renal function [HR 1.44; 95% CI 1.31–1.57; *P* < 0.001] in over-all AKI patients. Nevertheless, results illustrated that furosemide was not associated with reduced in-hospital mortality in patients with AKI stage 0–1 defined by UO criteria, AKI stage 2–3 according to SCr criteria, and in those with acute-on-chronic (A-on-C) renal injury.

**Conclusions:**

Furosemide administration was associated with improved short-term survival and recovery of renal function in critically ill patients with AKI. Furosemide was especially effective in patients with AKI UO stage 2–3 degree. However, it was not effective in those with AKI SCr stage 2–3 and chronic kidney disease. The results need to be verified in randomized controlled trials.

## Background

Acute kidney injury (AKI) is common in critically ill patients and carries a high morbidity and mortality rate. In clinical practice, diuretics, in particular loop diuretics, are often used to prevent AKI by increasing the urine output (UO) [1, 2]. Furosemide is the most common loop diuretic used in critically ill patients, and numerous clinical trials have been designed to evaluate the effectiveness of it in AKI. Some studies found that furosemide exhibited neutral or deleterious effects in AKI treatment [2–4]. In contrast, several studies have suggested that furosemide might reduce the need for renal replacement therapy (RRT) and attenuate the severity of AKI [5, 6]. So, the effect of furosemide on outcomes of critically ill patients with AKI is still controversial and needs to be further investigated in large-scale studies. We hypothesized that furosemide administration was associated with decreased mortality in critically ill patients with AKI.

Current guideline recommends defining AKI based on serum creatinine (SCr) increase and oliguria, and staging AKI by the worse of the two parameters [7]. To explore the effect of diuretics on outcomes of AKI, studies often included patients using both UO and SCr criteria [2, 8]. However, Kellum and colleagues in a study of 23,866 AKI patients found that the mortality of patients with AKI defined by UO criteria was much lower than those staged according to both UO and SCr criteria [9]. Studies also found that the patient’s UO criterion for AKI did not usually match well with the patient’s respective SCr criterion [10, 11]. As oliguria is the main reason for furosemide use in clinical practice, oliguric AKI should be considered as a single population in evaluating the effect of diuretics on outcomes in AKI patients. In addition, it has been noticed that the association between poor outcomes and furosemide was more frequently reported in cohorts with higher SCr (> 3.0 mg/dl) while insignificant in patients with mild AKI (< 2.0 mg/dl) [12]. The result suggested that the curative effect of furosemide on AKI patients may be influenced by the increased degree of SCr. The second aim of this study was to examine the association of furosemide use and mortality outcome among patients with different AKI stages by UO or SCr criteria or both.

## Materials and methods

### Sources of data

The data of the present study were collected from a large critical care database named Multiparameter Intelligent Monitoring in Intensive Care Database III (MIMIC III). MIMIC III is a publicly and freely available database which is well described in previous papers [13, 14]. In brief, MIMIC III database contains ICU patient data from the Beth Israel Deaconess Medical Center (single) between 2001 and 2012. This database was approved by the Institutional Review Boards (IRB) of the Massachusetts Institute of Technology (MIT). After successfully completing the National Institutes of Health (NIH) Web-based training course and the Protecting Human Research Participants examination (no. 7574829), we were given the permission to extract data from MIMIC III.

### Population selection criteria

Patients meeting criteria for AKI following the Kidney Disease: Improving Global Outcomes (KDIGO) criteria on admission were considered eligible for study inclusion. KDIGO criteria are as follows [7]: increase in SCr to ≥ 1.5 times baseline must have occurred within the prior 7 days; or a ≥ 0.3 mg/dl increase in SCr occurred within 48 h; or urine volume < 0.5 ml/kg/h for 6 h or more. Minimum of the SCr values available within the 7 days before admission was used as the baseline SCr [15, 16]. When the pre-admission SCr was not available, the first SCr measured at admission was used as the baseline SCr [17]. AKI stages were defined by both SCr and the volume of UO during the first 48 h after ICU admission. AKI stages defined by SCr (AKI^Cre^) or UO (AKI^UO^) alone were recorded respectively. If patients were admitted multiple times, only the first stay was analyzed. Patients with age < 18 years old, and those who were discharged or died within 48 h after ICU admission were excluded. Patients were also excluded from the study if they received diuretic drugs 48 h after ICU admission or if more than 5% of the potential risk variables for death were missing.

### Data collection and definitions

The data on the first day of ICU admission were extracted from MIMIC III using Structured Query Language (SQL) with Navicat Premium (version 12.0.28) including age, gender, ethnicity, admission type, comorbidities, simplified Acute Physiology Score II (SAPSII), sequential organ failure assessment (SOFA) score, mean arterial pressure (MAP), SCr level, use of vasopressors and inotropes, daily fluid input, fluid balance, fluid types, cardiac surgery, RRT, and mechanical ventilation. Sepsis was defined as life-threatening organ dysfunction caused by a dysregulated host response to infection (sepsis 3.0). In the present study, patients with documented or suspected infection plus an acute increase of ≥ 2 SOFA points were recorded as sepsis. The estimated glomerular filtration rate (eGFR) was calculated using the modification of diet in renal disease (MDRD) formula [18].

The information on diuretic drugs used including drug names, dose, route, start time, and end time were also collected. A Python script was written by us for calculating the dose of furosemide and for collecting the administration route of it. Total furosemide dose was defined as the intravenous administration plus 0.5 × oral dose. In evaluating the dose-dependent effect of furosemide on the outcomes of AKI patients, the data of furosemide dose were presented as milligram per kilogram per day.

In the present study, all variables had less than 25% missing values. Single imputation was used to impute missing values in variables including MAP, serum creatinine and eGFR on admission, and weight and the volume of UO on ICU discharge (see additional file 1: Table S1).

### Endpoints

In-hospital mortality was the primary endpoint. 90-day mortality, recovery of renal function, length of stay (LOS) in hospital, and ICU were considered as secondary outcomes. Recovery of kidney function was defined as being discharged from ICU with SCr below 1.5 times the baseline value and normal UO (> 0.5 ml/kg/h for 24 h on discharge).

### Statistical analysis

Continuous variables in the present study were all expressed by median [interquartile range (IQR)] and the differences between groups were identified with the Mann-Whitney test because of their non-normal distribution. Categorical variables were expressed as the number and percentage, and comparisons between groups were made using the chi-square test or Fisher’s exact test as appropriate.

To estimate the association between furosemide administration and outcomes among critically ill patients with AKI, propensity-score matching was performed in our study by a greedy nearest neighbor matching using a caliper of 0.2 standard deviations of the logit of the estimated propensity score. Patients were matched in a 1:1 ratio, such that each patient who was treated with furosemide within 48 h after ICU admission was matched to 1 patient without diuretic treatment. Standardized mean difference (SMD) was calculated to evaluate the efficiency of PSM in reducing the differences between the two groups.

Cox regression model was used for estimating the relationships between administration of furosemide and mortality outcomes adjusting for confounding variables selected based on *p* value < 0.05 in univariate analysis and potential confounders judged by clinical expertise of our team and was used for estimating the relationships between quintiles of furosemide dose and mortality. Impact of furosemide use on the recovery of renal function was estimated using logistic regression model adjusting for age, gender, and SAPSII score. Linear regression was used to evaluate the association between furosemide use and length of stay, and the hazard ratios (HR) were calculated using the formula HR = *e*^βi^.

Stratification analysis was conducted to explore whether the association between furosemide administration and in-hospital mortality differed across various subgroups classified by different AKI severity based on SCr or UO criteria or both, chronic kidney disease, acute respiratory distress syndrome (ARDS), heart failure, and sepsis. The relationship between the daily dose of furosemide and in-hospital mortality was also evaluated, and the analysis was performed in the population after PSM matching.

Statistical analysis was performed using IBM SPSS Statistics version 22.0 (IBM, Armonk, NY, USA) and R 3.5.3 software for windows and Python 3.7.3. A *P* value < 0.05 was considered statistically significant.

## Results

### Basic characteristics

**Fig. 1:**
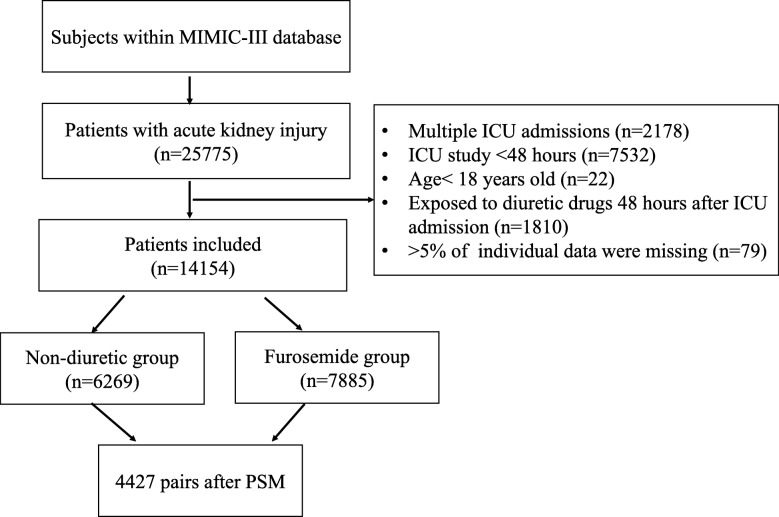
Flowchart of Included Patients. MIMIC III: Multiparameter Intelligent Monitoring in Intensive Care Database III; ICU: intensive care unit; PSM: propensity-score matching

During the study period, 25,775 critically ill patients were admitted with AKI. After excluding the patients according to the exclusion criteria, 14,154 eligible patients were enrolled. Seven thousand eight hundred eighty-five patients were exposed to furosemide within the first 48 h after ICU admission, and 6269 patients did not receive diuretics (Fig. 1).

As shown in Table 1, there were significant differences in AKI stages, ethnicity, and admission types between the furosemide group and the non-diuretic group. The mean age was significantly higher, and the levels of MAP were significantly lower among furosemide-treated patients on admission. The levels of SCr were significantly higher and eGFR was lower in the non-diuretic group when compared with the furosemide group. Vasopressors and inotropes use were more common in the furosemide group. Patients with heart failure, acute lung edema, and those who underwent cardiac surgery were more likely to be given furosemide. The volume of daily fluid input and the incidence of colloid use were higher among furosemide-treated patients. The proportion of patients with positive fluid balance in the furosemide group was greater than that in non-diuretic group.

**Table 1:** Baseline characteristics between groups before matching

Variables	Non-diuretic group *n* = 6269	Furosemide group *n* = 7885	*P* value	SMD
AKI stage, *n* (%)			< 0.001	0.132
Stage 1	1953(31.2)	2293(29.1)		
Stage 2	2715(43.3)	3908(49.6)		
Stage 3	1601(25.5)	1684(21.4)		
Age	67.8 (54.7,78.9)	69.9 (59.2,79.4)	< 0.001	0.167
Gender, male, *n* (%)	3503 (55.9)	4419 (56.0)	0.844	0.003
Ethnicity, *n* (%)			< 0.001	0.125
White	4271 (68.1)	5758 (73.0)		
Black	721 (11.5)	648 (8.2)		
Others	1277 (20.4)	1479(18.8)		
Admission type, *n* (%)			< 0.001	0.424
Elective surgery	421 (6.7)	1575 (20.0)		
Emergency surgery	1073 (17.1)	1554 (19.7)		
Medical	4775 (76.2)	4756 (60.3)		
Co-morbidities, *n* (%)				
CKD	639 (10.2)	661 (8.4)	< 0.001	0.062
Diabetes	1969 (31.4)	2637 (33.4)	0.011	0.043
Heart failure	1774 (28.3)	3733 (47.3)	< 0.001	0.401
Chronic lung disease	1151 (18.4)	1806 (22.9)	< 0.001	0.112
Chronic liver disease	649 (10.4)	558 (7.1)	< 0.001	0.116
Cancer	419 (6.7)	314 (4.0)	< 0.001	0.120
Hypertension	3431 (54.7)	4985 (63.2)	< 0.001	0.173
Sepsis	2484 (39.6)	3607 (45.7)	< 0.001	0.124
ARDS	1413 (22.5)	1857 (23.5)	0.156	0.024
Acute lung edema	27 (0.4)	91 (1.2)	< 0.001	0.082
Cardiac surgery	252(4.0)	1755(22.3)	< 0.001	0.561
Mechanical ventilation, no. (%)	3016 (48.1)	5654 (71.7)	< 0.001	0.496
RRT, *n* (%)	383 (6.1)	293 (3.7)	< 0.001	0.111
MAP^a^	79.3 (68.0,93.0)	78.0 (68.0,90.0)	< 0.001	0.082
Vasopressors use, *n* (%)	2616 (41.7)	4832 (61.3)	< 0.001	0.399
Inotropes use, *n* (%)	301(4.8)	1135(14.4)	< 0.001	0.330
Fluid balance			< 0.001	0.144
Volume (ml)	− 615(− 1500,450)	− 393(− 1440,1025)		
Positive, *n* (%)	1968(31.4)	3058(38.8)		
Daily fluid input (ml)	201 (0, 580)	289 (51,756)	< 0.001	0.084
Colloid input	378 (6.0)	969 (12.3)	< 0.001	0.218
Serum creatinine^a^	114.9(79.6203.3)	106.1(79.6168.0)	< 0.001	0.191
eGFR, ml/min/1.73 m^2b^	49.9(23.5,82.0)	56.4(30.9,81.9)	< 0.001	0.097
SAPSII score^c^	39 (30,50)	39 (32,48)	0.745	0.010

*Abbreviations*: *CKD* chronic kidney diseases, *ARDS* acute respiratory distress syndrome, *RRT* renal replacement therapy, *IQR* interquartile range, *MAP* mean arterial pressure, *eGFR* estimated glomerular filtration rate, *SAPSII* Simplified Acute Physiology Score II, *SMD* standardized mean difference

^a^The first values during the first day after ICU admission were recorded

^b^ eGFR was calculated using MDRD formula

^c^ SAPSII score was calculated within the first 24 h after the ICU admission using the value associated with the greatest severity of illness

### Relationship between furosemide and outcomes

Cox proportional hazard model was used to examine the difference in mortality outcomes between the two groups. In pre-matched cohort, furosemide use was associated with reduced in-hospital mortality (HR 0.63; 95% CI 0.58–0.69; *P* < 0.001) and 90-day mortality (HR 0.66; 95% CI 0.61–0.70; *P* < 0.001) after adjusting for possible confounding factors associated with mortality (Table 2 and Additional file 1: Table S2). Using the logistic regression model, the impact of furosemide use on the recovery of renal function was estimated, and we found that furosemide use was also associated with an increased chance of renal function recovery (HR 1.29; 95% CI 1.21–1.38; *P <* 0.001). Nevertheless, furosemide use was associated with longer length of stay (LOS) in ICU and hospital (Table 2).

**Table 2:** Association between furosemide use and clinical outcomes in critically ill patients with acute kidney injury

	Non-diuretic group	Furosemide group	*P* value	HR	Lower 95% CI	Upper 95% CI
Pre-matched cohort	*n* = 6269	*n* = 7885				
Primary outcome						
In-hospital mortality, *n* (%)^a^	1363(21.7)	1001(12.7)	< 0.001	0.63	0.58	0.69
Secondary outcomes						
90-day mortality, n (%)^a^	1981(31.6)	1673(21.2)	< 0.001	0.66	0.61	0.70
Recovery of renal function, n (%)^b^	2939(46.9)	4209(53.4)	< 0.001	1.29	1.21	1.38
Length of ICU stay, [median (IQR)]^c^	3.91(2.8, 6.8)	4.13(2.9, 7.4)	0.003	1.44	1.28	1.62
Length of hospital stay, [median (IQR)]^c^	9.57(6.0, 16.2)	10.08(6.8, 16.3)	0.013	1.37	1.12	1.68
Post-matched cohort	*n* = 4427	n = 4427				
Primary outcome						
In-hospital mortality, *n* (%)^a^	974(22.0)	635(14.3)	< 0.001	0.67	0.60	0.74
Secondary outcomes						
90-day mortality, *n* (%)^a^	1442(32.6)	1054(23.8)	< 0.001	0.69	0.64	0.75
Recovery of renal function, *n* (%)^b^	2620(59.2)	2991(67.6)	< 0.001	1.44	1.31	1.57
Length of ICU stay, [median (IQR)]^c^	4.1(2.9, 7.1)	4.1(2.9, 7.2)	0.221	1.28	0.89	1.62
Length of hospital stay, [median (IQR)]^c^	10.0(6.4, 16.9)	10.5(6.5, 16.4)	0.032	1.71	1.04	2.85

*Abbreviations*: *HR* hazard ratio, *CI* confidence interval, *ICU* intensive care unit, *IQR* interquartile range

^a^ Cox regression was used for estimating the impact of furosemide use on mortality outcomes adjusting for confounding variables selected based on *P* value < 0.05 in univariate analysis

^b^ Recovery from acute kidney injury was defined as being discharged from ICU with serum creatinine below 1.5 times the baseline value and normal urine output (> 0.5 ml/kg/h). Impact of furosemide use on the recovery of renal function was estimated using the logistic regression model

^c^ Linear regression was used to evaluate the association between furosemide use and length of stay. HR was calculated using the formula HR = *e*^βi^

In PSM, 4427 patients who received furosemide were matched with 4427 patients who did not receive diuretics. In Table S3 (see Additional file 1), the matched patient characteristics were compared, and the standardized mean differences (SMD) for all the individual covariates were provided. After matching, the baseline profiles were well balanced between the two groups with SMDs that were less than 10% for all variables (Table S3 and Additional file 1: Figure S1). Similar to the results in the pre-matched model, furosemide was associated with reduced in-hospital mortality (HR 0.67; 95% CI 0.61–0.74; *P* < 0.001), and it was also associated with improved 90-day survival (HR 0.69; 95% CI 0.64–0.75; *P* < 0.001) after PSM (Table 2). The results showed that the recovery of renal function was promoted by furosemide in AKI patients (HR 1.44; 95% CI 1.31–1.57; *P* < 0.001) (Table 2). Additionally, furosemide was also associated with increased LOS in hospital (HR 1.71; 95% CI 1.04–2.85; *P* = 0.032) (Table 2).

When taking the dose of furosemide into consideration, we found that receiving ≤1.10 mg/kg/day was associated with a reduced risk of in-hospital mortality when compared with the non-diuretic group (Table S4). High-dose furosemide (> 1.10 mg/kg/day) did not improve the outcome of critically ill patients with AKI (HR 0.870; 95% CI 0.742–1.020; *P* = 0.085) (Additional file 1: Table S4).

### Subgroup analysis

**Fig. 2:**
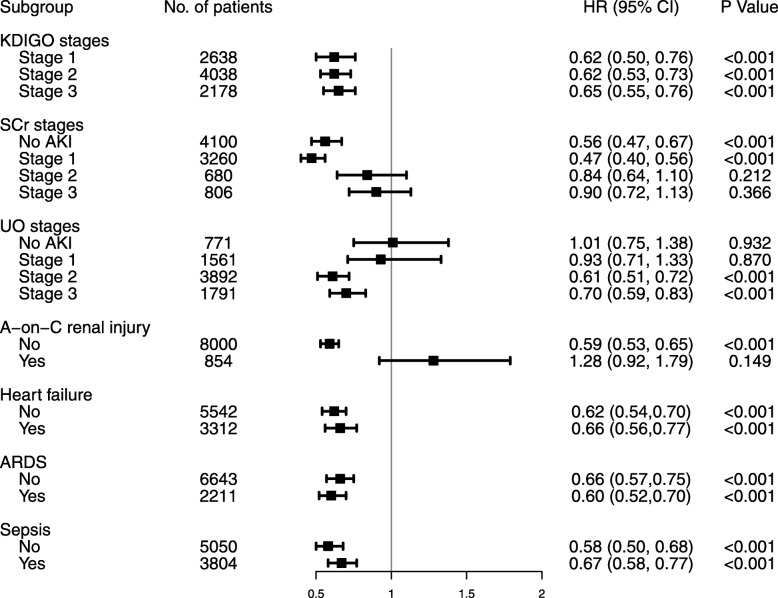
The association between furosemide administration and in-hospital mortality in subgroups. AKI: acute kidney injury; KDIGO: kidney disease improving global outcomes; SCr: serum creatinine; UO: urine output; A-on-C renal injury: acute-on-chronic renal injury. ARDS: Acute respiratory distress syndrome; HR: hazard ratio; CI: confidence interval

The number of patients in each subgroup was showed in Table S5 (see Additional file 1). As shown in Table S6 (see Additional file 1) and Fig. 2, the furosemide was associated with improved in-hospital mortality in patients with AKI stage 1 to 3 according to the KDIGO criteria. When AKI stage was defined by SCr criteria (AKI^SCr^), the improved outcome was observed in patients with AKI^SCr^ stage 0 or 1 but not in those with AKI^SCr^ stage 2 or 3. Interestingly, when AKI stage was defined by UO criteria alone (AKI^UO^), furosemide was associated with improved in-hospital mortality in patients with AKI^UO^ stage 2 or 3 but not in those with AKI^UO^ stage 0 or 1. When the analysis was restricted to patients with CKD, furosemide was not associated with improved in-hospital outcomes. Other subgroups were not significant.

The characteristics of patients with different stages of AKI were shown in Table S7. There were 23.2% and 36.9% of cases with positive fluid balance in patients with AKI^UO^ stage 0–1 and AKI^UO^ stage 2–3, respectively. Additionally, the proportion of patients with positive fluid balance in AKI^SCr^ stage 2–3 was greater than that in AKI^SCr^ stage 0–1 (*P* < 0.001) (Additional file 1: Table S7).

## Discussion

Our results demonstrated furosemide administration was associated with reduced short-term mortality in critically ill patients with AKI. The results of the present cohort also suggest that furosemide may promote renal function recovery. Consistent with our study, a previous controlled study found that furosemide appeared to increase renal function recovery rates [19]. Theoretically, furosemide may prevent AKI by decreasing the GFR and tubular workload, and then reduce renal medullary oxygenation [20, 21]. Additionally, some scholars assumed that furosemide could act as renal vasodilators [22]. However, these theoretical arguments were proved in experimental conditions, but not in clinical practice. The results of several previous studies and meta-analysis did not support the use of furosemide in AKI patients [3–5, 23], and the KDIGO clinical practice guideline for AKI also suggested against the use of diuretics in AKI management [7]. A recent meta-analysis of 28 randomized controlled trials found that furosemide administration was not associated with increased mortality in patients with or at risk for AKI, and it may reduce mortality when used as a preventive measure [24]. Unfortunately, the severity and fluid status of AKI was not included in the study [24]. It is necessary to further analyze the influence of furosemide on outcomes in different subsets of AKI patients.

Oliguria still represents one of the two main criteria for the diagnosis of AKI, and it is also the main reason for using diuretics. In our cohort, there were 7244 (82%) patients with AKI according to UO criteria alone. Positive fluid balance is an expected complication of oliguria in AKI patients. Furosemide is helpful in the management of fluid overload. The results of the present study showed that positive fluid balance was more common in patients with AKI oliguria stage 2–3, and beneficial effect of furosemide on in-hospital mortality was especially observed in this population. A multicenter ICU study also found that, in patients with a higher fluid balance and a lower volume of urine output, diuretic use was associated with better survival [25]. So, the beneficial effects of furosemide on mortality in oliguric AKI patients may be mediated by fluid balance.

Unlike SCr criteria, defining AKI with the UO criteria may be too liberal because several studies illustrated that AKI defined by UO was not an independent predictor of mortality [26, 27], and oliguria was not always indicative of a reduced GFR or tubular dysfunction [28]. In AKI patients with an increase in SCr by two or more times the baseline, we found that furosemide use was not associated with a significant decrease in in-hospital mortality, even in those accompanied by UO less than 0.5 ml/kg for more than 12 h (data not shown). In fact, Shen et al. have noticed that the effect of furosemide on mortality in AKI patients depends on the levels of SCr, and that the association between increased risk of death and furosemide was more frequently reported in cohorts with higher SCr [3, 8, 12]. So, urine volume output and the levels of SCr need to be considered together when using diuretics to treat AKI.

AKI patients with prior CKD, named acute-on-chronic (A-on-C) renal injury, is commonly seen in clinical practice with an incidence ranging from 13 to 35 per 100 AKI patients [29, 30]. In the pre-matched cohort, the incidence of A-on-C renal injury was 9.2%. More and more evidence suggest that epidemiological characteristics and outcomes of patients with A-on-C renal injury differ from AKI patients without prior CKD [29–31]. Nevertheless, the effect of furosemide on outcomes of A-on-C renal injury remains exclusive. In the present study, we first found that furosemide use was not associated with improved short-term survival in patients with A-on-C renal injury.

Our study has important limitations. Most notably, the MIMIC III database used in the present study only contains the data of critically ill patients admitted between 2001 and 2012. The definition of AKI has evolved in 2012. Thus, it is possible that our cohort is not exactly meeting the new definition of AKI. However, we have tried to identify AKI patients according to the newest criteria for AKI diagnosis (KDIGO criteria). Second, changes in treatment strategies for critically ill patients, including metabolism and nutritional support strategies and methods of mechanical ventilation, may influence the outcomes of AKI. However, there is no direct evidence which shows that these factors associated with the efficacy of furosemide in critically ill patients with AKI, and the clinical practice of AKI have not changed significantly in the past two decades. Third, only AKI patients receiving furosemide within 48 h after admission were included for analysis in our study. So, the effect of delayed use of furosemide on outcomes of AKI needs to be further investigated. Fourth, although the sample size of subgroups of the present study is relatively larger than previous studies, multiple subgroup analysis may also increase the risk of false-positive findings. Fifth, it was a retrospective design. Despite careful propensity score matching, residual confounding cannot be fully excluded. So, the risk of confounding factors should be taken into consideration when interpreting the results. Finally, this was a single-center study. The results need to be validated by multicenter trials.

## Conclusions

The use of furosemide was associated with reduced short-term mortality and improved recovery of renal function in critically ill patients with AKI. Furosemide was especially effective in patients with AKI oliguria stage 2–3 degree, but not in those with AKI SCr stage 2–3 and chronic kidney disease. Our results may be helpful for the rational use of furosemide in critically ill patients with AKI. The results need to be verified in the future by multicenter randomized controlled trials.

## Supplementary information

**Additional file 1: Table S1.** Missing number (%) for risk variables and outcome variables. **Table S2.** Potential risk variables for in-hospital death. **Table S3.** Comparisons after propensity score matching. **Table S4.** dose-response relationship between furosemide administration and in-hospital mortality. ***Table S5.*** The number of patients in subgroups. **Table S6.** The association between furosemide use and in-hospital mortality in subgroups. **Table S7.** the characteristics of patients with different stages of AKI. **Figure S1.** Standardized mean difference (SMD) of variables before and after propensity score matching.

## Data Availability

The datasets used in the present study are available from the first author and corresponding authors on reasonable request.

## Abbreviations

AKI: Acute kidney injury
A-on-C renal injury: Acute-on-chronic renal injury
CI: Confidence interval
D-AKI: Dialysis requiring acute kidney injury
eGFR: Estimated glomerular filtration rate
HR: Hazard ratio
ICU: Intensive care unit
ICU: Intensive care unit
IQR: Interquartile range
KDIGO: Kidney disease: improving global outcomes
MAP: Mean arterial pressure
MDRD: Modification of diet in renal disease
MIMIC III: Multiparameter Intelligent Monitoring in Intensive Care Database III
RRT: Renal replacement therapy
SAPSII: Simplified Acute Physiology Score II
SCr: Serum creatinine
SMD: Standardized mean difference
UO: Urine output
