# Supplementary material for: Association between furosemide administration and outcomes in critically ill patients with acute kidney injury
Source: Crit Care. 2020 Mar 4;24:75. doi: 10.1186/s13054-020-2798-6 (PMC7057586; doi:10.1186/s13054-020-2798-6)
Supplement: Supplementary file 1 — Additional file 1: Table S1. Missing number (%) for risk variables and outcome variables. Table S2. Potential risk variables for in-hospital death. Table S3. Comparisons after propensity score matching. Table S4. dose-response relationship between furosemide administration and in-hospital mortality. Table S5. The number of patients in subgroups. Table S6. The association between furosemide use and in-hospital mortality in subgroups. Table S7. the characteristics of patients with different stages of AKI. Figure S1. Standardized mean difference (SMD) of variables before and after propensity score matching. [file 13054_2020_2798_MOESM1_ESM.docx]

**Table S1. Missing number (%) for risk variables and outcome variables**

| **Risk variables** | **Missing number (%)** |
| --- | --- |
| AKI stage | 0(0) |
| Age | 0(0) |
| Gender, male | 0(0) |
| Ethnicity | 0(0) |
| Admission type | 0(0) |
| Chronic kidney disease | 0(0) |
| Diabetes | 0(0) |
| Heart failure | 0(0) |
| Chronic lung disease | 0(0) |
| Chronic liver disease | 0(0) |
| Metastatic cancer | 0(0) |
| Hypertension | 0(0) |
| Sepsis | 0(0) |
| ARDS | 0(0) |
| Acute lung edema | 0(0) |
| MAP^a^ | 34(0.2%) |
| Vasopressors use | 0(0) |
| Mechanical ventilation | 0(0) |
| RRT | 0(0) |
| Cardiac surgery | 0(0) |
| Inotropes | 0(0) |
| Fluid balance | 0(0) |
| Volume of fluid input | 0(0) |
| Colloid input | 0(0) |
| Serum creatinine^a^ | 23(0.2%) |
| eGFR^a^ | 23(0.2%) |
| SAPSII score^b^ | 0(0) |
| **Outcome variables** |  |
| Weight^c^ | 843(5.9%) |
| Urine output^c^ | 1227(8.7%) |
| Serum creatinine^c^ | 15(0.1%) |
| Hospital admission time | 0(0) |
| Hospital discharge time | 0(0) |
| Hospital admission time | 0(0) |
| ICU discharge time | 0(0) |
| Death time | 0(0) |

Abbreviations: ARDS: Acute respiratory distress syndrome; RRT: renal replacement therapy; IQR: interquartile range; MAP: mean arterial pressure; eGFR: estimated glomerular filtration rate; SAPSII: Simplified Acute Physiology Score II; ICU: intensive care unit.

^a^ The first values during the first day after ICU admission were recorded;

^b^ SAPSII score was calculated within the first 24 h after the ICU admission using the value associated with the greatest severity of illness;

c The values on ICU discharge were recorded.

**Table S2. Potential risk variables for in-hospital death**

| **Variables** | **HR** | **95% CI** | **P value** |
| --- | --- | --- | --- |
| **AKI stage (per 1 stage increase)** | 1.131 | 1.055~1.212 | <0.001 |
| **Age (per 1 year increase)** | 1.134 | 1.058~1.215 | <0.001 |
| **Male** | 1.065 | 0.994~1.141 | 0.074 |
| **White face** | 0.928 | 0.833~1.034 | 0.176 |
| **Emergency surgery** | 0.985 | 0.999~0.870 | <0.001 |
| **Co-morbidities** |  |  |  |
| Chronic kidney disease | 0.735 | 0.610~0.885 | 0.001 |
| Diabetes | 0.871 | 0.779~0.974 | 0.016 |
| Heart failure | 1.087 | 0.974~1.213 | 0.137 |
| Chronic lung disease | 0.875 | 0.772~0.991 | 0.035 |
| Chronic liver disease | 1.343 | 1.162~1.552 | <0.001 |
| Cancer | 1.533 | 1.297~1.811 | <0.001 |
| Hypertension | 0.823 | 0.751~0.921 | <0.001 |
| Sepsis | 1.107 | 0.999~1.227 | 0.052 |
| ARDS | 1.758 | 1.580~1.957 | <0.001 |
| Acute lung edema | 1.314 | 0.799~2.162 | 0.282 |
| **Cardiac surgery** | 0.580 | 0.417~0.808 | 0.001 |
| **MAP (per 1 mmHg increase)^a^** | 0.999 | 0.996~1.001 | 0.412 |
| **Vasopressors use** | 1.448 | 1.288~1.628 | <0.001 |
| **Mechanical ventilation** | 1.096 | 0.972~1.237 | 0.136 |
| **RRT** | 1.343 | 1.148~1.570 | <0.001 |
| **Inotropes** | 1.543 | 1.322~1.800 | <0.001 |
| **Fluid balance (per 100ml increase)** | 1.006 | 1.004~1.007 | <0.001 |
| **Positive fluid balance** | 1.227 | 1.110-1.356 | <0.001 |
| **Daily fluid input (per 100ml increase)** | 1.023 | 1.018~1.013 | <0.001 |
| **Colloid** | 0.942 | 0.789~1.111 | 0.475 |
| **Serum creatinine^a^ (per 10 mmol/L increase)** | 1.003 | 1.001~1.005 | 0.006 |
| **eGFR with 1 unit increase** | 0.994 | 0.992~1.000 | <0.001 |
| **SAPSII^b^ with 1 score increase** | 1.033 | 1.029~1.037 | <0.001 |

Abbreviations: ARDS: Acute respiratory distress syndrome; RRT: renal replacement therapy; IQR: interquartile range; MAP: mean arterial pressure; eGFR: estimated glomerular filtration rate; SAPSII: Simplified Acute Physiology Score II; SMD: standardized mean difference

^a^ The first values during the first day after ICU admission were recorded.

^b^ SAPSII score was calculated within the first 24 h after the ICU admission using the value associated with the greatest severity of illness.

**Table S3. Comparisons after propensity score matching**

| **Variables** | **Non-diuretic group**  **n=4427** | **Furosemide group**  **n=4427** | **P** | **SMD** |
| --- | --- | --- | --- | --- |
| **AKI stage, n (%)** |  |  | 0.855 | 0.012 |
| **Stage1** | 1312(29.6) | 1236(30.0) |  |  |
| **Stage2** | 2015(45.5) | 2023(45.7) |  |  |
| **Stage3** | 1100(24.8) | 1078(24.4) |  |  |
| **Age** | 69.0(56.0,79.4) | 68.6(57.5,78.7) | 0.886 | 0.020 |
| **Gender, male,** **n (%)** | 2456 (55.5) | 2445 (55.2) | 0.814 | 0.005 |
| **Ethnicity,** **n (%)** |  |  | 0.581 | 0.022 |
| White | 3080 (69.6) | 3119 (70.5) |  |  |
| Black | 468(10.6) | 442 (10.0) |  |  |
| Others | 879 (19.9) | 866(19.6) |  |  |
| **Admission type,** **n (%)** |  |  | 0.818 | 0.013 |
| Elective surgery | 388 (8.8) | 405 (9.1) |  |  |
| Emergency surgery | 790 (17.8) | 785 (17.7) |  |  |
| Medical | 3249(73.4) | 3237 (73.1) |  |  |
| **Co-morbidities,** **n (%)** |  |  |  |  |
| CKD | 423 (9.6) | 431 (9.7) | 0.773 | 0.006 |
| Diabetes | 1432 (32.3) | 1420 (32.1) | 0.785 | 0.006 |
| Heart failure | 1651(37.3) | 1661 (37.5) | 0.826 | 0.005 |
| Chronic lung disease | 937 (21.2) | 927 (20.9) | 0.794 | 0.006 |
| Chronic liver disease | 418 (9.4) | 417(9.4) | 0.971 | 0.001 |
| Cancer | 257 (5.8) | 263 (5.9) | 0.786 | 0.006 |
| Hypertension | 2572 (58.1) | 2571 (58.1) | 0.983 | 0.001 |
| Sepsis | 1894 (42.8) | 1910 (43.1) | 0.731 | 0.007 |
| ARDS | 1131 (25.5) | 1080 (24.4) | 0.211 | 0.027 |
| Acute lung edema | 25 (0.6) | 29 (0.7) | 0.585 | 0.012 |
| **Cardiac surgery** | 251(5.7) | 253(5.7) | 0.927 | 0.002 |
| **Mechanical ventilation,**  **n (%)** | 2619 (59.2) | 2582 (58.3) | 0.424 | 0.017 |
| **RRT, n (%)** | 225 (5.1) | 220 (5.0) | 0.808 | 0.005 |
| **MAP ^a^** | 79.0(67.7,92.0) | 79.3(68.0,91.7) | 0.449 | 0.003 |
| **Vasopressors use, n (%)** | 2124 (48.0) | 2132 (48.2) | 0.865 | 0.004 |
| **Inotropes use, n (%)** | 288(6.5) | 305(6.9) | 0.470 | 0.015 |
| **Fluid balance**  **Volume (ml)**  **Positive, n (%)** | -562(-1460,535)  1462(33.0) | -575(-1550,610)  1477(33.4) | 0.500  0.752 | 0.014 |
| **Daily fluid input (ml)** | 203.0(0.0,574.1) | 205.5(0.0,592.4) | 0.194 | 0.022 |
| **Colloid input** | 317 (7.2) | 333 (7.5) | 0.514 | 0.014 |

**Table S3 (Continued)**

| **Variables** | **Non-diuretic group**  **n=4427** | **Furosemide group**  **n=4427** | **P** | **SMD** |
| --- | --- | --- | --- | --- |
| **Serum creatinine^a^** | 114.9(79.6,194.5) | 106.1(79.6,185.6) | 0.224 | 0.026 |
| **eGFR, ml/min/1.73 m2^b^** | 50.7(24.8,81.1) | 53.1(27.0,80.2) | 0.257 | 0.017 |
| **SAPSII score**^c^ | 40(30,50) | 39(32,48) | 0.945 | 0.010 |

Abbreviations: CKD: Chronic kidney diseases; ARDS: Acute respiratory distress syndrome; RRT: renal replacement therapy; IQR: interquartile range; MAP: mean arterial pressure; eGFR: estimated glomerular filtration rate; SAPSII: Simplified Acute Physiology Score II; SMD: standardized mean difference

^a^ The first values during the first day after ICU admission were recorded;

^b^ SAPSII score was calculated within the first 24 h after the ICU admission using the value associated with the greatest severity of illness.


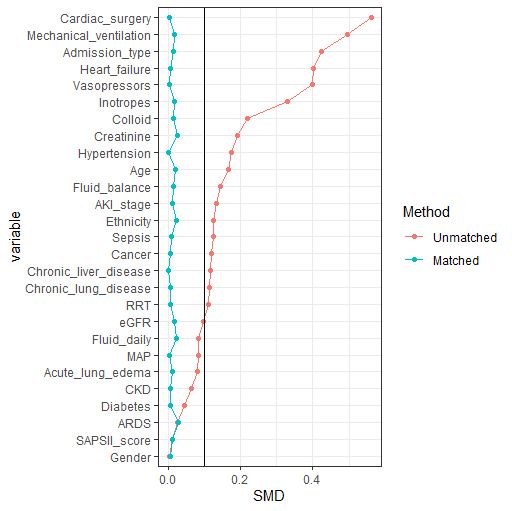


**Figure S1. Standardized mean difference (SMD) of variables before and after propensity score matching.**

Abbreviations: ARDS: Acute respiratory distress syndrome; RRT: renal replacement therapy; CKD: chronic kidney disease; IQR: interquartile range; MAP: mean arterial pressure; eGFR: estimated glomerular filtration rate; SAPSII: Simplified Acute Physiology Score II; SMD: standardized mean difference;

**Table S4. dose-response relationship between furosemide administration and in-hospital mortality**

| **Variables** | **HR** | **95% CI** | **P value** |
| --- | --- | --- | --- |
| **Furosemide dose (mg/kg/d)** |  |  |  |
| **Without furosemide** | 1.000 |  |  |
| **<0.24** | 0.536 | 0.440-0.653 | <0.001 |
| **0.25-0.38** | 0.480 | 0.388-0.593 | <0.001 |
| **0.39-0.61** | 0.667 | 0.554-0.804 | <0.001 |
| **0.62-1.10** | 0.600 | 0.498-0.722 | <0.001 |
| **>1.10** | 0.870 | 0.742-1.020 | 0.085 |
| **AKI stage (per 1 stage increase)** | 1.128 | 1.053-1.208 | 0.001 |
| **Age (per 1 year increase)** | 1.014 | 1.010-1.017 | <0.001 |
| **Emergency surgery** | 0.918 | 0.803-1.051 | 0.214 |
| **Co-morbidities** |  |  |  |
| Chronic kidney disease | 0.730 | 0.607-0.879 | 0.001 |
| Chronic liver disease | 1.346 | 1.167-1.552 | <0.001 |
| Cancer | 1.526 | 1.294-1.799 | <0.001 |
| Hypertension | 0.833 | 0.752-0.922 | <0.001 |
| ARDS | 1.807 | 1.631-2.001 | <0.001 |
| **Cardiac surgery** | 1.807 | 1.631-2.001 | <0.001 |
| **Vasopressors use** | 1.420 | 1.268-1.590 | <0.001 |
| **Inotropes use** | 1.572 | 1.349-1.831 | <0.001 |
| **RRT** | 1.503 | 1.286-1.756 | <0.001 |
| **Fluid balance**  **Volume (pre 100 ml increase)**  **Positive** | 1.000  0.947 | 0.998-1.002  0.853-1.051 | 0.905  0.308 |
| **Daily fluid input (pre 100 ml increase)** | 1.019 | 1.014-1.024 | <0.001 |
| **Serum creatinine**  **(pre 10 mmol/L increase)** | 0.994 | 0.989-1.001 | 0.056 |
| **eGFR with 1 unit increase** | 0.994 | 0.992-0.997 | <0.001 |
| **SAPSII score with 1 score increase** | 1.020 | 1.017-1.024 | <0.001 |

Abbreviations: RRT: renal replacement therapy; IQR: interquartile range; MAP: mean arterial pressure; eGFR: estimated glomerular filtration rate; SAPSII: Simplified Acute Physiology Score II.

**Table S5. The number of patients in subgroups**

| **Subgroups** | **Non-diuretic group**  **n=4427** | **Furosemide group**  **n=4427** |
| --- | --- | --- |
| **KDIGO stages, n(%)** |  |  |
| Stage 1 | 1312(29.6) | 1326(30.0) |
| Stage 2 | 2015(45.5) | 2023(45.7) |
| Stage 3 | 1100(24.8) | 1078(24.4) |
| ^a^**SCr stages, n(%)** |  |  |
| No AKI | 2099(47.4) | 2001(45.2) |
| Stage 1 | 1604(36.2) | 1656(37.4) |
| Stage 2 | 351(7.9) | 329(7.4) |
| Stage 3 | 368(8.3) | 438(9.9) |
| ^b^**UO stages, n(%)** |  |  |
| No AKI | 393(8.9) | 378(8.5) |
| Stage 1 | 834(18.8) | 727(16.4) |
| Stage 2 | 1928(43.6) | 1964(44.4) |
| Stage 3 | 898(20.3) | 893(20.2) |
| **A-on-C renal injury, n(%)** |  |  |
| No | 4004(90.4) | 3996(90.3) |
| Yes | 423(9.6) | 431(9.7) |
| **Heart failure, n(%)** |  |  |
| No | 2776(62.7) | 2766(62.5) |
| Yes | 1651(37.3) | 1661(37.5) |
| **ARDS, n(%)** |  |  |
| No | 3296(74.5) | 3347(75.6) |
| Yes | 1131(25.5) | 1080(24.4) |
| **Sepsis, n(%)** |  |  |
| No | 2533(57.2) | 2517(56.9) |
| Yes | 1894(42.8) | 1910(43.1) |

Abbreviations: AKI: acute kidney injury; KDIGO: kidney disease: improving global outcomes; SCr: serum creatinine; UO: urine output; A-on-C renal injury: acute-on-chronic renal injury. ARDS: Acute respiratory distress syndrome

**^a^**8 lacking information of SCr stages were censored.

**^b^**839 patients lacking information of UO stages were censored.

**Table S6. The association between furosemide use and in-hospital mortality in subgroups**

| **Subgroups** | **No. patients** | **HR (95% CI)** | **P value** |
| --- | --- | --- | --- |
| **KDIGO stages** |  |  |  |
| Stage 1 | 2638 | 0.62 (0.50, 0.76) | <0.001 |
| Stage 2 | 4038 | 0.62 (0.53, 0.73) | <0.001 |
| Stage 3 | 2178 | 0.65 (0.55, 0.76) | <0.001 |
| ^a^**SCr stages** |  |  |  |
| No AKI | 4100 | 0.56 (0.47, 0.67) | <0.001 |
| Stage 1 | 3260 | 0.47 (0.40, 0.56) | <0.001 |
| Stage 2 | 680 | 0.84 (0.64, 1.10) | 0.212 |
| Stage 3 | 806 | 0.90 (0.72, 1.13) | 0.366 |
| ^b^**UO stages** |  |  |  |
| No AKI | 771 | 1.01 (0.75, 1.38) | 0.932 |
| Stage 1 | 1561 | 0.93 (0.71, 1.33) | 0.870 |
| Stage 2 | 3892 | 0.61 (0.51, 0.72) | <0.001 |
| Stage 3 | 1791 | 0.70 (0.59, 0.83) | <0.001 |
| **A-on-C renal injury** |  |  |  |
| No | 8000 | 0.59 (0.53, 0.65) | <0.001 |
| Yes | 854 | 1.28 (0.92, 1.79) | 0.149 |
| **Heart failure** |  |  |  |
| No | 5542 | 0.62 (0.54,0.70) | <0.001 |
| Yes | 3312 | 0.66 (0.56,0.77) | <0.001 |
| **ARDS** |  |  |  |
| No | 6643 | 0.66 (0.57,0.75) | <0.001 |
| Yes | 2211 | 0.60 (0.52,0.70) | <0.001 |
| **Sepsis** |  |  |  |
| No | 5050 | 0.58 (0.50, 0.68) | <0.001 |
| Yes | 3804 | 0.67 (0.58, 0.77) | <0.001 |

Abbreviations: HR: hazard ratio; CI: confidence interval. AKI: acute kidney injury; KDIGO: kidney disease: improving global outcomes; SCr: serum creatinine; UO: urine output; A-on-C renal injury: acute-on-chronic renal injury. ARDS: Acute respiratory distress syndrome

**^a^**8 lacking information of SCr stages were censored.

**^b^**839 patients lacking information of UO stages were censored.

**Table S7. the characteristics of patients with different stages of AKI**

| **Variables** | **UO criteria^a^** | | | **SCr criteria^b^** | | |
| --- | --- | --- | --- | --- | --- | --- |
|  | **Stage 0-1**  **n=2332** | **Stage 2-3**  **n=5683** | **P value** | **Stage 0-1**  **n=7360** | **Stage 2-3**  **n=1486** | **P value** |
| **Age** | 67.2 (55.0-78.0) | 69.4 (57.9-79.6) | <0.001 | 69.3 (57.5-79.5) | 66.0 (53.8-76.5) | <0.001 |
| **Male** | 1374 (58.9%) | 3057 (53.8%) | <0.001 | 4084 (55.5%) | 814 (54.8%) | 0.615 |
| **White face** | 1607 (68.9%) | 4033 (71.0%) | 0.067 | 5157 (70.1%) | 1035 (69.7%) | 0.748 |
| **Emergency surgery** | 375 (16.1%) | 1070 (18.8%) | 0.004 | 1319 (17.9%) | 254 (17.1%) | 0.446 |
| **Co-morbidities** |  |  |  |  |  |  |
| CKD | 201 (8.6%) | 433 (7.6%) | 0.132 | 601 (8.2%) | 253 (17.0%) | <0.001 |
| Diabetes | 738 (31.6%) | 1806 (31.8%) | 0.908 | 2368 (32.2%) | 480 (32.3%) | 0.924 |
| Heart failure | 809 (34.7%) | 2112 (37.2%) | 0.037 | 2685 (36.5%) | 625 (42.1%) | <0.001 |
| Chronic lung disease | 470 (20.2%) | 1200 (21.1%) | 0.336 | 1537 (20.9%) | 327 (22.0%) | 0.333 |
| Chronic liver disease | 182 (7.8%) | 547 (9.6%) | 0.010 | 644 (8.8%) | 190 (12.8%) | <0.001 |
| Cancer | 126 (5.4%) | 344 (6.1%) | 0.261 | 400 (5.4%) | 120 (8.1%) | <0.001 |
| Hypertension | 1325 (56.8%) | 3322 (58.5%) | 0.178 | 4312 (58.6%) | 825 (55.5%) | 0.029 |
| Sepsis | 948 (40.7%) | 2463 (43.3%) | 0.027 | 3105 (42.2%) | 696 (46.8%) | <0.001 |
| ARDS | 529 (22.7%) | 1437 (25.3%) | 0.014 | 1738 (23.6%) | 473 (31.8%) | <0.001 |
| Acute lung edema | 10 (0.4%) | 32 (0.6%) | 0.450 | 37 (0.5%) | 17 (1.1%) | 0.004 |
| **Cardiac surgery** | 137 (5.9%) | 337 (5.9%) | 0.924 | 444 (6.0%) | 60 (4.0%) | 0.002 |
| **MAP** | 80.0 (68.0-92.0) | 79.0 (68.0-92.0) | 0.449 | 80.0 (68.0-92.0) | 77.0 (66.0-89.5) | <0.001 |
| **Vasopressors use** | 1098 (47.1%) | 2789 (49.1%) | 0.105 | 3451 (46.9%) | 800 (53.8%) | <0.001 |
| **Mechanical ventilation** | 1373 (58.9%) | 3408 (60.0%) | 0.365 | 4301 (58.4%) | 898 (60.4%) | 0.155 |
| **RRT** | 83 (3.6%) | 316 (5.6%) | <0.001 | 287 (3.9%) | 158 (10.6%) | <0.001 |

**Table S7 (Continued)**

| **Variables** | **UO criteria** | | | **SCr criteria** | | |
| --- | --- | --- | --- | --- | --- | --- |
|  | **Stage 0-1**  **n=2332** | **Stage 2-3**  **n=5683** | **P value** | **Stage 0-1**  **n=7360** | **Stage 2-3**  **n=1486** | **P value** |
| **Inotropes** | 126 (5.4%) | 393 (6.9%) | 0.012 | 454 (6.2%) | 139 (9.4%) | <0.001 |
| **Fluid balance**  **Volume**  **Positive** | -1287 (-2295,0)  541 (23.2%) | -449 (-1245,816)  2099 (36.9%) | <0.001  <0.001 | -740 (-1615,413)  2256 (30.7%) | 0 (-687,1454)  681 (45.8%) | <0.001  <0.001 |
| **Daily fluid input** | 201.4 (0.0-614.8) | 212.6 (0.0-576.2) | 0.257 | 200.5 (0.0-573.0) | 228.2 (0.0-621.5) | 0.023 |
| **Colloid** | 133 (5.7%) | 467 (8.2%) | <0.001 | 516 (7.0%) | 134 (9.0%) | 0.007 |
| **Serum creatinine** | 106.1 (70.7-168.0) | 106.1 (79.6-176.8) | 0.018 | 106.1 (79.6-159.1) | 203.3 (106.1-353.6) | <0.001 |
| **eGFR** | 58.8 (31.7-85.0) | 53.6 (27.9-81.5) | <0.001 | 57.0 (31.8-83.3) | 24.8 (13.4-53.5) | <0.001 |
| **SAPSII** | 38.0 (30.0-47.0) | 40.0 (32.0-50.0) | <0.001 | 39.0 (31.0-48.0) | 44.0 (35.0-55.0) | <0.001 |

Abbreviations: SCr: serum creatinine; UO: urine output; CKD: Chronic kidney diseases; ARDS: Acute respiratory distress syndrome; RRT: renal replacement therapy; IQR: interquartile range; MAP: mean arterial pressure; eGFR: estimated glomerular filtration rate; SAPSII: Simplified Acute Physiology Score II

**^a^**839 patients lacking information of UO stages were censored.

**^b^**8 patients lacking information of SCr stages were censored.
